# Supplementary material for: The Lipidomics of Spermatozoa and Red Blood Cells Membrane Profile of Martina Franca Donkey: Preliminary Evaluation
Source: Animals (Basel). 2022 Dec 20;13(1):8. doi: 10.3390/ani13010008 (PMC9817730; doi:10.3390/ani13010008)
Supplement: Supplementary file 1 [file animals-13-00008-s001.zip › animals-2070208-supplementary.pdf]

Supplementary Table S1. The detailed characteristics of the donkeys involved in the study

N	Age (years)	BCS
1	2	4/5
2	4	4/5
3	3	3/5
4	12	3/5
5	19	3/5
6	2	4/5
7	4	4/5
8	13	3/5
9	3	3/5
10	12	3/5
11	5	3/5
12	19	3/5
13	2	4/5
14	4	4/5
15	13	3/5
16	19	3/5
17	3	3/5
18	19	3/5
19	2	4/5
20	19	3/5
21	4	4/5
22	19	3/5
23	5	3/5
24	19	3/5
25	5	3/5
26	19	3/5

Supplementary Table S2: The materials were used as received and are listed in the following table along with the corresponding suppliers:

Materials	Company
n-Hexane 95%	TITOLCHIMICA, Pontecchio Polesine (Ro) Italy
Methyl alcohol HPLC	TITOLCHIMICA Pontecchio Polesine (Ro) Italy
Chloroform extra pure 99.5%	TITOLCHIMICA Pontecchio Polesine (Ro) Italy
PBS pH 7,4 RS	Carlo Erba, Milan (Italy)
Polar Lipid Mixture (quantitative)	MATREYA LLC State College, PA, USA
non-Polar Lipid Mixture B (quantitative)	MATREYA LLC State College, PA,USA
Phosphatidylserine	MATREYA LLC State College, PA, USA
L-α-Phosphatidylcholine	Merck, Darmstadt, Germany
ALUGRAM Xtra sheets 200x200mm	Carlo Erba, Milan Italy
Potassium hydroxide, pellets RPE - For analysis	Carlo Erba, Milan Italy
Sodium sulfate anhydrous RS - For anhydrification	Carlo Erba, Milan Italy
C14:0 – myristic acid methyl ester	Merck, Darmstadt Germany
C16:0 – palmitic acid methyl ester	Merck, Darmstadt Germany
C16:1 – palmitoleic acid methyl ester	Merck, Darmstadt Germany
C18:0 – stearic acid methyl ester	Supelco, Bellefonte, PA, USA
9c, C18:1 – oleic acid methyl ester	Merck, Darmstadt, Germany
11c, C18:1 – vaccenic acid methyl ester	Supelco, Bellefonte, PA ,USA
LA omega-6 – C18:2 – linoleic acid methyl ester	Merck, Darmstadt Germany
DGLA omega-6 – C20:3 dihomogammalinolenic acid methyl ester	Merck, Darmstadt Germany
ARA omega-6 – C20:4 – arachidonic acid methyl ester	Merck, Darmstadt Germany
EPA omega-3 – C20:5 – eicosapentaenoic acid methyl ester	Supelco, Bellefonte, PA, USA
DPA omega-6 – C22:5 – Docosapentenoic acid methyl ester	Merck, Darmstadt Germany
DHA omega-3 – C22:6 – docosahexaenoic acid methyl ester	Merck, Darmstadt Germany
Supelco 37 component FAME mix	Supelco, Bellefonte, PA, USA

GC analysis of FAME – Calibration procedure

For this study we chose to study a cluster of 12 fatty acids, which also corresponds to chromatographic peak areas >97%. This cluster consists of: 3 saturated fatty acids (SFA: myristic, palmitic and stearic acids); 3 monounsaturated fatty acids (MUFA, palmitoleic, oleic and cis-vaccenic acids); 4 polyunsaturated fatty acids omega-6 (PUFA, linoleic, dihomo-gamma linolenic, arachidonic, docosapentenoic acids); 2 polyunsaturated fatty acids omega-3 (PUFA, eicosapentaenoic and docosahexaenoic acids) as shown in Table 1 of the main text.

Taking into account the previously reported benchmark for membrane fatty acid profile [15] we proceeded with the quantitation of the fatty acids was carried out by calibration procedures, for which the following protocol has been followed:

initially a *n*-hexane (HPLC grade, Titolchimica) 5mM solution of stearic acid methyl ester (2 mg in 1340 μL) was prepared and 1μl was directly injected to the Agilent 7890B GC system equipped with a flame ionization detector and a DB-23 (50%-Cyanopropyl)-methylpolysiloxane capillary column (60 m, 0.25 mm i.d., 0.25 μm film thickness). The following oven conditions were established to be kept for all the analyses: the initial temperature was 165 °C, held for 3 min, followed by an increase of 1 °C/min up to 195 °C, held for 40 min, followed by a second increase of 10 °C/min up to 240 °C, held for 10 min. The carrier gas was hydrogen, held at a constant pressure of 16.482 psi. The injections were repeated in triplicates.

The second round of injections for calibration was then performed with 0.5 mM solution of the same fatty acid methyl ester (taking 100μL of the initial solution and diluting with 900μL of *n*-hexane), injecting 1 μL as previously described for triplicates.

The same protocol was carried out using dilutions of 0.05mM, 0.005mM and 0.0005mM of stearic acid methyl ester.

In all the injections a calibration curve was created using the software of the GC equipment (Agilent 8890B GC system). Using the concentration of 0.0005mM for methyl stearate, the corresponding peak area was detectable but not quantifiable, indicating this concentration as the limit of detection (LOD) of the specific GC system (<0.5nM). The same protocol has been followed for all the fatty acids of the cohort.

Reference FAMEs				
**SFA**	**C14:0**	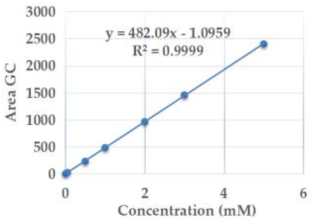	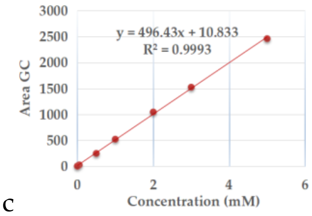<br>c	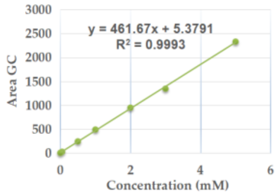
	**C16:0**	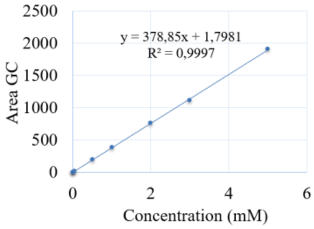	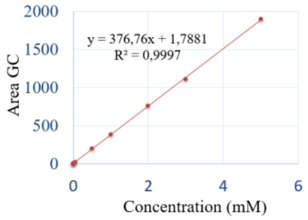	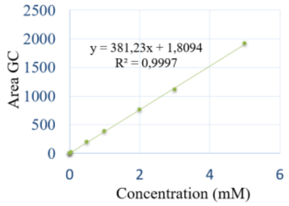
	**C18:0**	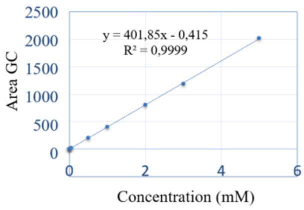	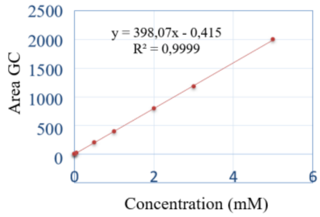	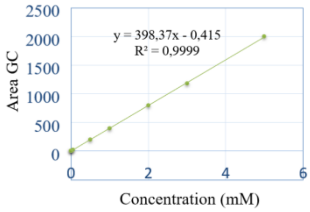
**MUFA**	**9c,C16:1**	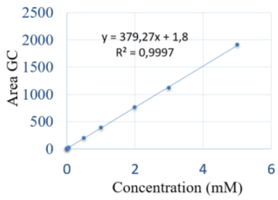	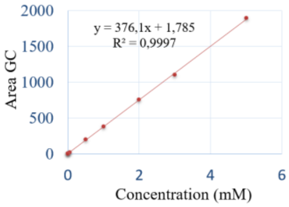	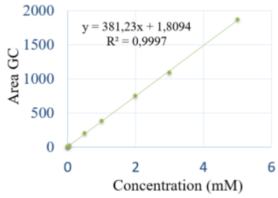
	**9c,C18:1**	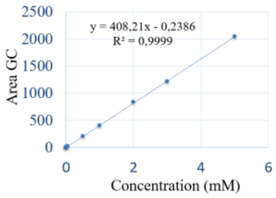	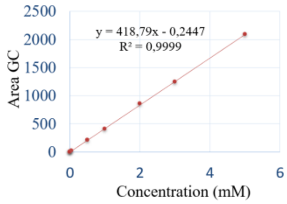	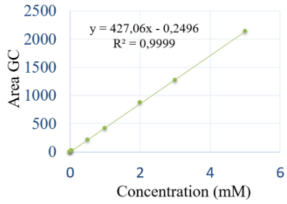
	**11c,C18:1**	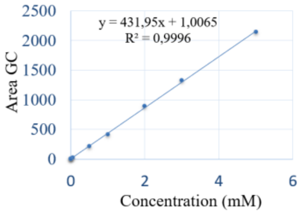	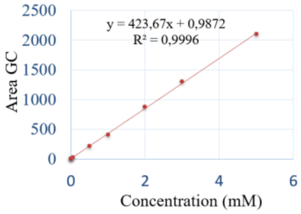	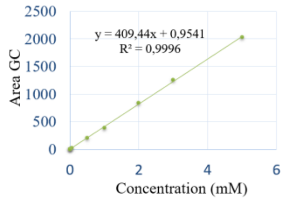
**PUFA omega-3**	**EPA**	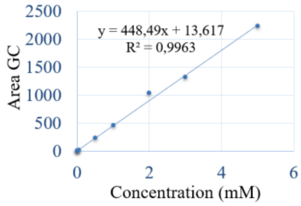	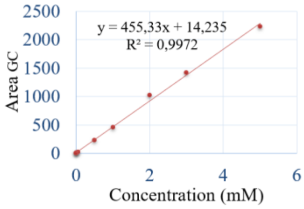	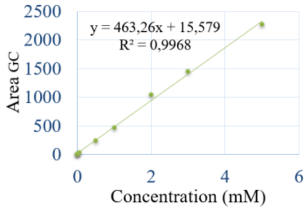

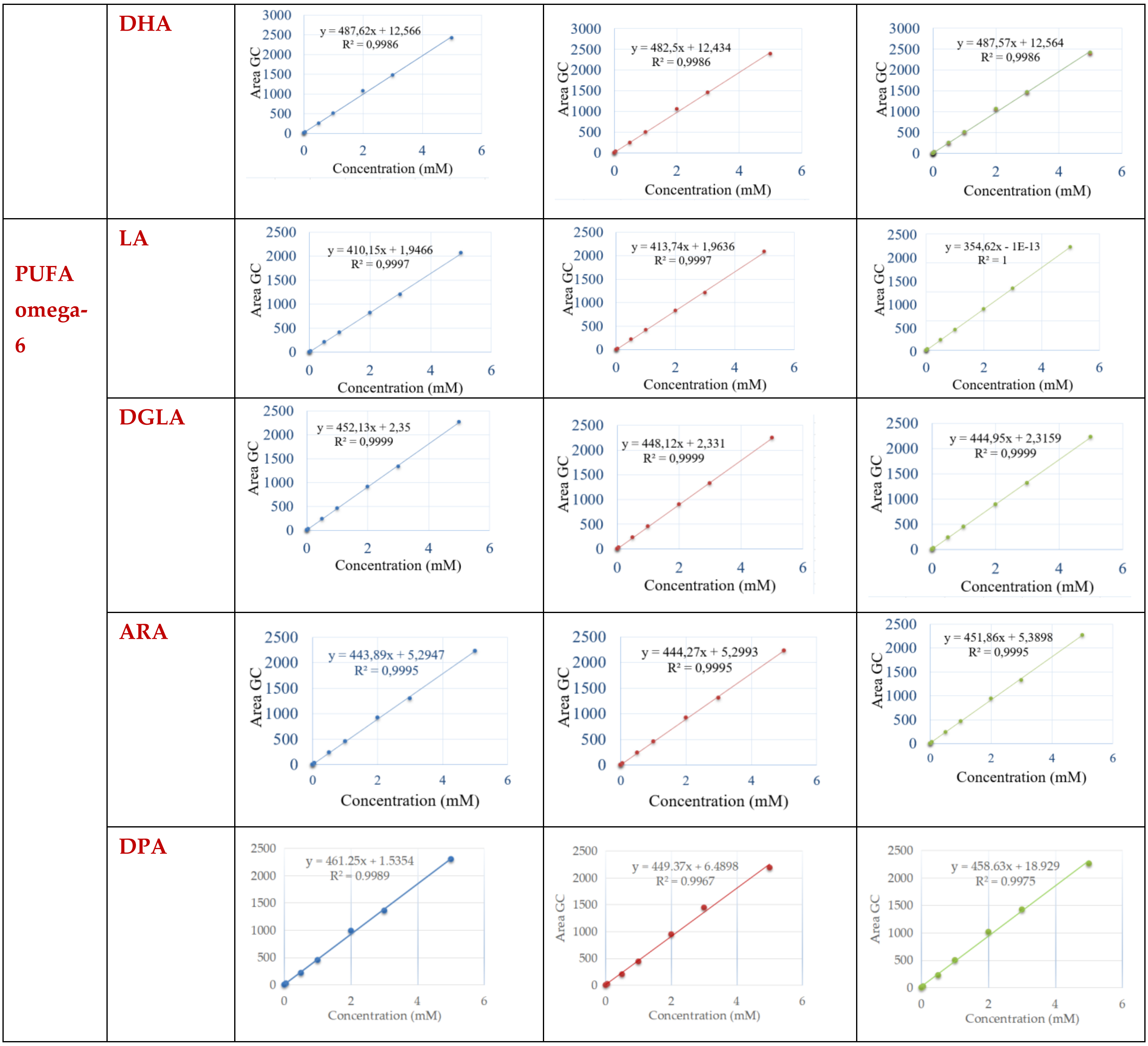

Supplementary Figure S1. Calibration curves of the 12 fatty acids chosen as representatives of the SFA, MUFA and PUFA families present in the erythrocyte membrane phospholipids.

Supplementary Table S3: Individual values of sperm parameters of healthy donkeys (n=26)

Don-keys' ID	Reaction time (min)	Total vol-ume (ml)	Gel free vol-ume (ml)	Concentration (mln/ml)	Dead (mln/ml)	Motil-ity (%)	Progressive Motility (%)
1	11	65	55	139	19	89	59
2	15	50	45	885	109	78	45
3	35	50	40	518	155	86	42
4	14	50	30	1600	108	88	56
5	4	30	28	492	133	82,7	56,5
6	6	65	60	385	96,5	84,8	59,9
7	11	55	50	1429	122	95,3	39,4
8	35	60	55	430	93	58,8	17,6
9	5	65	60	495	74,25	60	36
10	8	70	42,5	727	196,8	89,8	35,3
11	11	50	40	658	119	92	52,3
12	8	50	35	278	58,3	59,5	50
13	12	60	50	165	30	68	46
14	10	55	45	352	82	91	32
15	13	50	45	304	78	89	57
16	5	65	60	130	34	68	56
17	9	45	25	271	56,7	20	13
18	10	40	25	299	77,5	44	25
19	10	60	50	450	56	31	14
20	8	20	10	525	134	47	4
21	6	70	60	581	116	44	35
22	15	60	50	258	63	36	12
23	8	40	32	1122	90	37	26
24	8	30	20	194	83	30	21
25	45	40	30	650	88	69	41
26	5	45	35	120	27	57	51

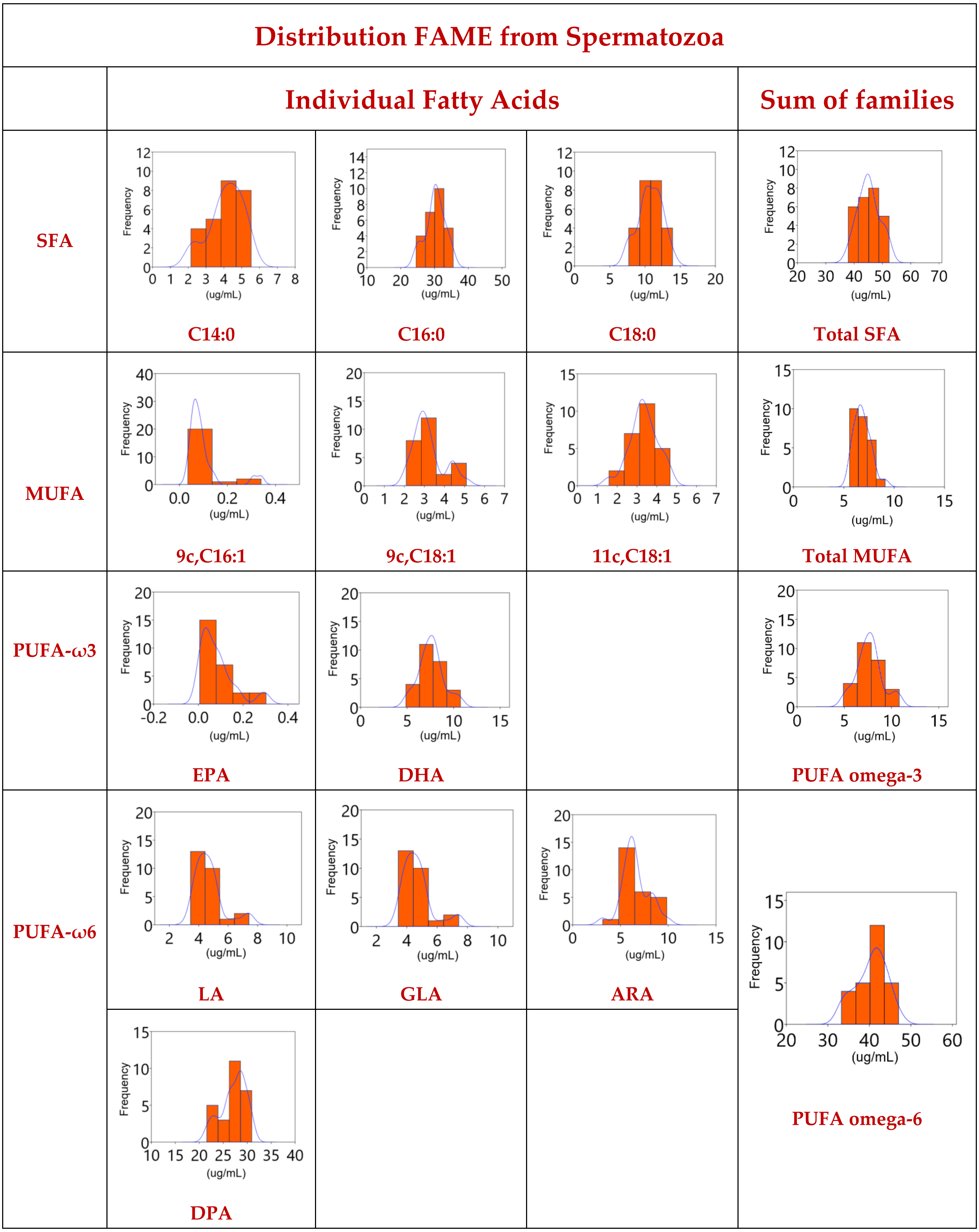

Supplementary Figure S2. Distribution of the values in the population of 26 healthy donkeys for each of the fatty acids obtained from spermatozoa membranes (data are reported in Table 2 in the main text). Each member of the fatty acid family is given in a row, the last column being the sum of the corresponding fatty acid family.

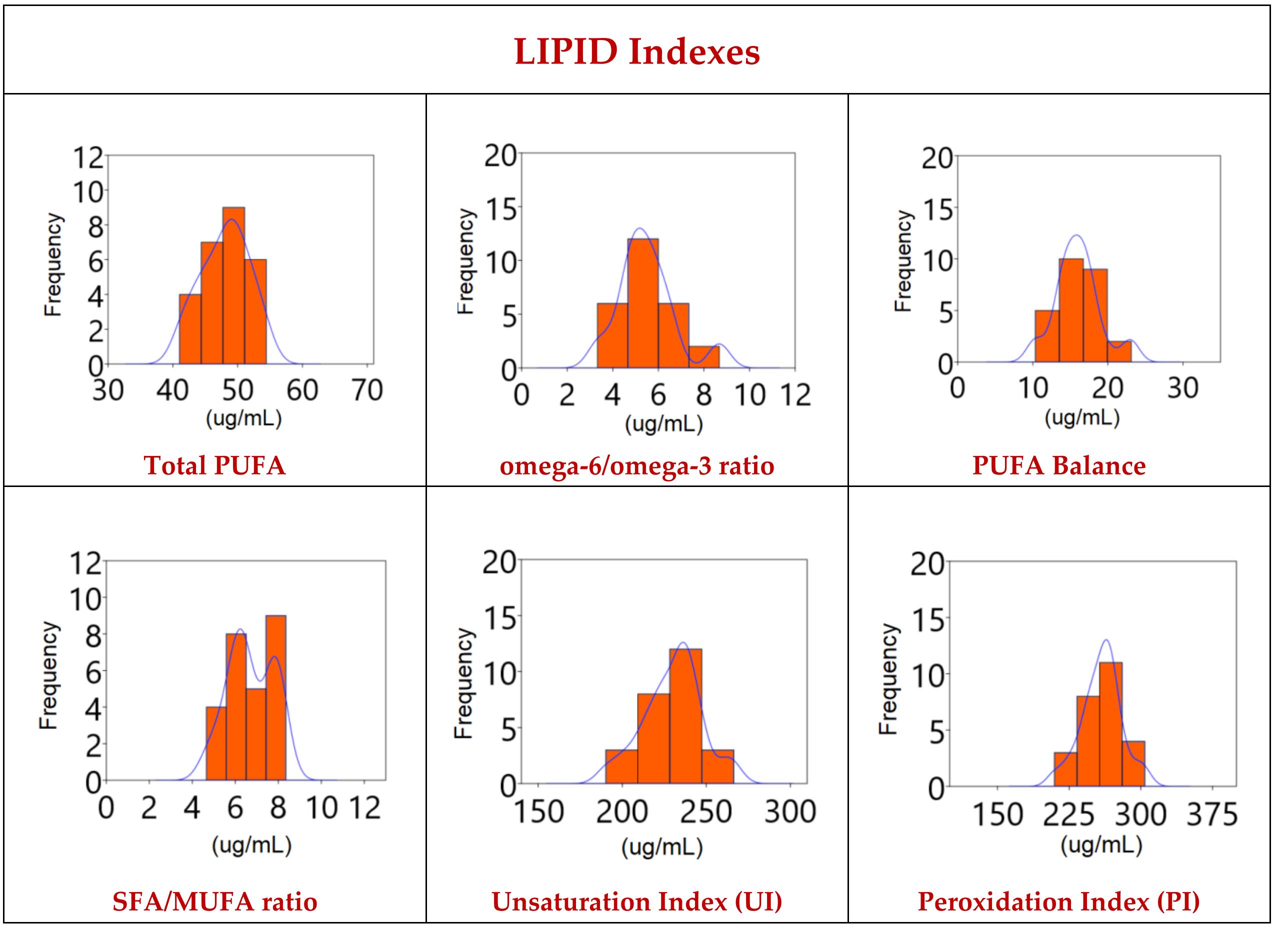

Supplementary Figure S3. Distribution of the values in the population of the 26 healthy donkeys for the lipid indexes obtained from spermatozoa membranes (data are reported in Table 2 in the main text). 1st row: total PUFA, omega-6/omega-3 and PUFA balance ratios; 2nd row: SFA/MUFA ratio, unsaturation and peroxidation indexes.

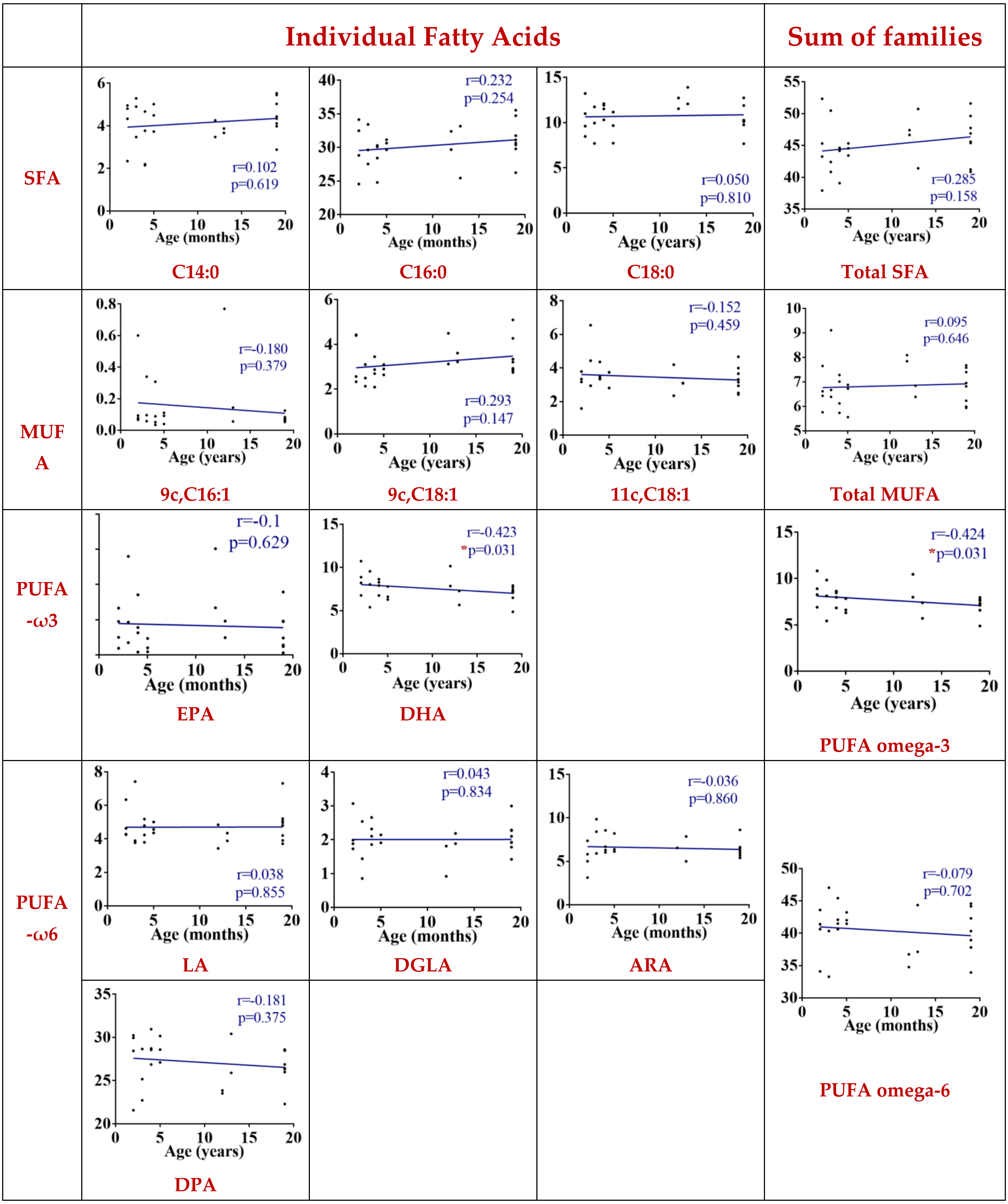

Supplementary Figure S4. Spearman correlation with linear regression and parameters for the 26 healthy donkeys using age and each fatty acid type and family obtained from spematozoa membranes (data are reported in Table 2 in the main text). Each member of the fatty acid family is given in a row, the last column being the sum of the corresponding fatty acid family.

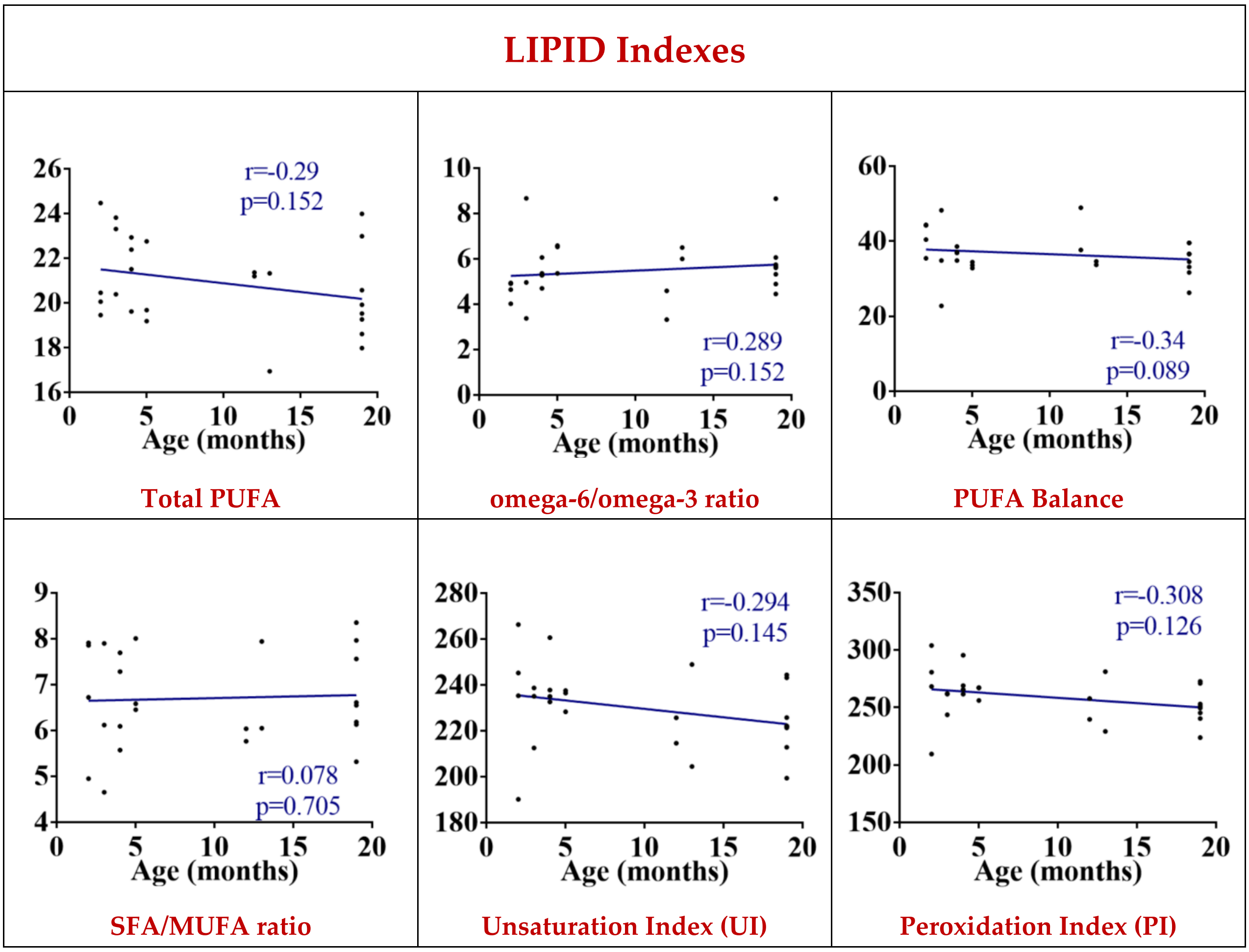

Supplementary Figure S5. Spearman correlation with linear regression and parameters for the 26 healthy donkeys using age and lipid indexes obtained from spermatozoa membranes (data are reported in Table 2 in the main text). 1st row: total PUFA, omega-6/omega-3 and PUFA balance ratios; 2nd row: SFA/MUFA ratio, unsaturation and peroxidation indexes.

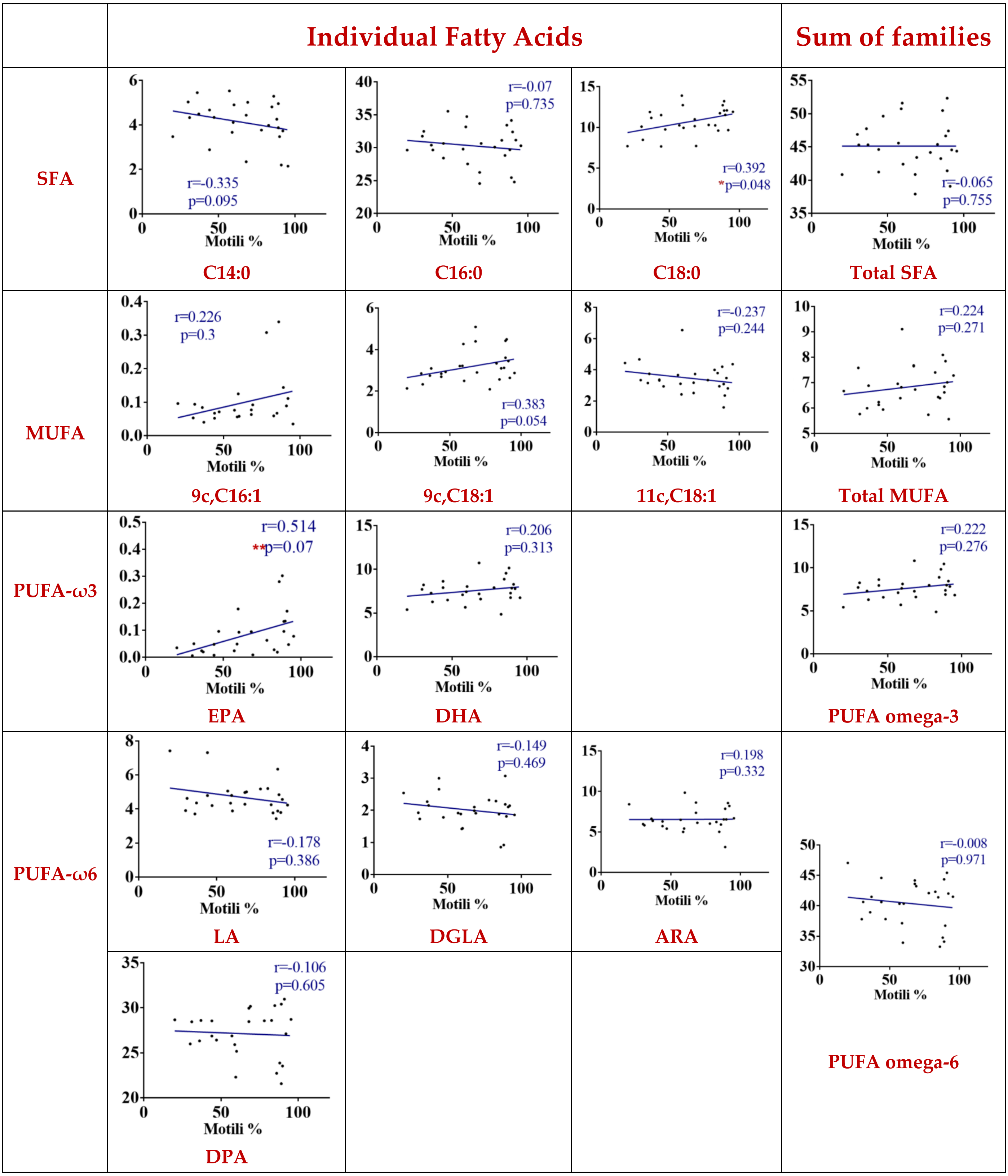

Supplementary Figure S6. Spearman correlation with linear regression and parameters for the 26 healthy donkeys using motility (%) and each fatty acid type and family obtained from spermatozoa membranes (data are reported in Table 2 in the main text). Each member of the fatty acid family is given in a row, the last column being the sum of the corresponding fatty acid family.

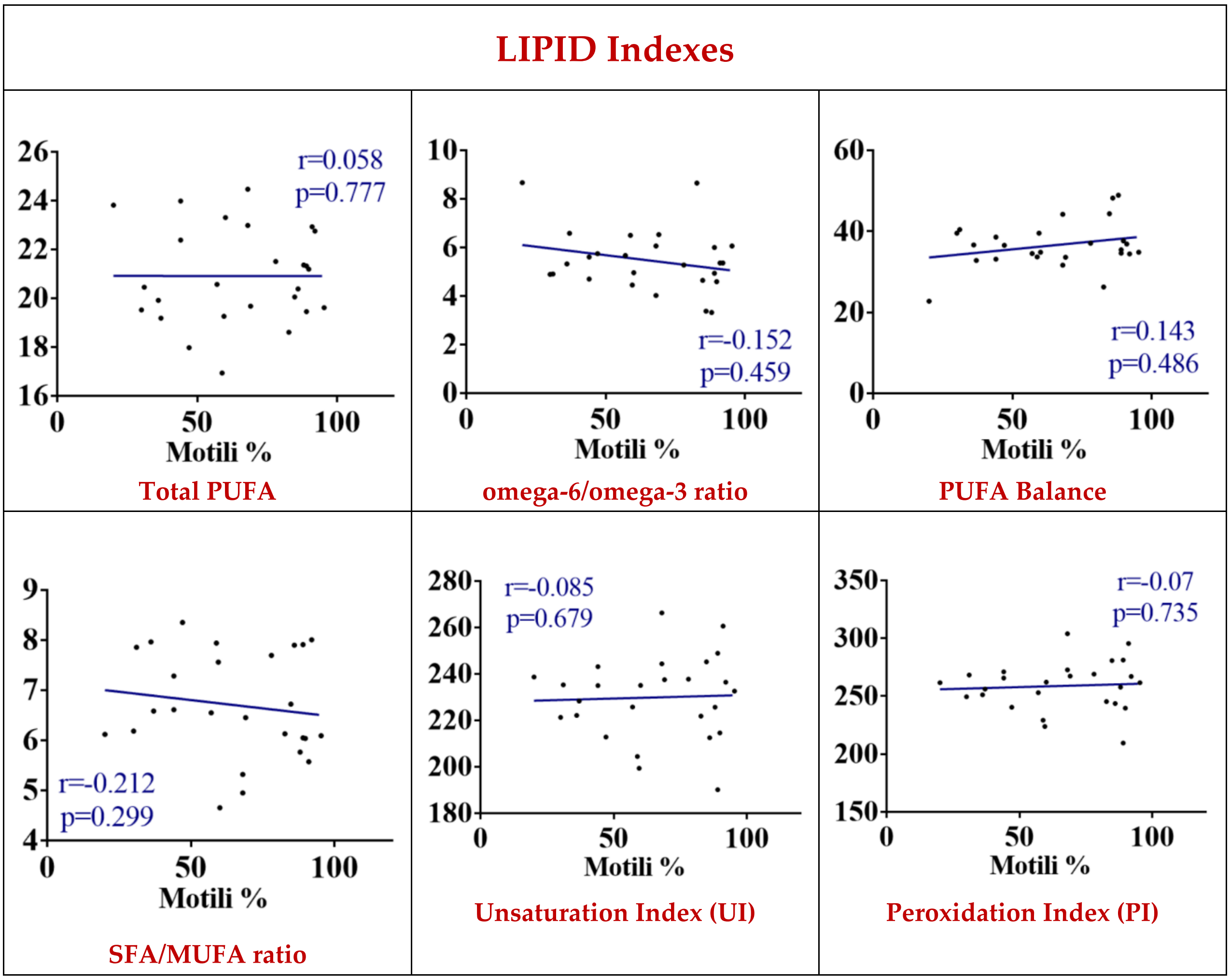

Supplementary Figure S7. Spearman correlation with linear regression and parameters for the 26 healthy donkeys using motility (%) and lipid indexes obtained from spermatozoa membranes (data are reported in Table 2 in the main text). 1st row: total PUFA, omega-6/omega-3 and PUFA balance ratios; 2nd row: SFA/MUFA ratio, unsaturation and peroxidation indexes.

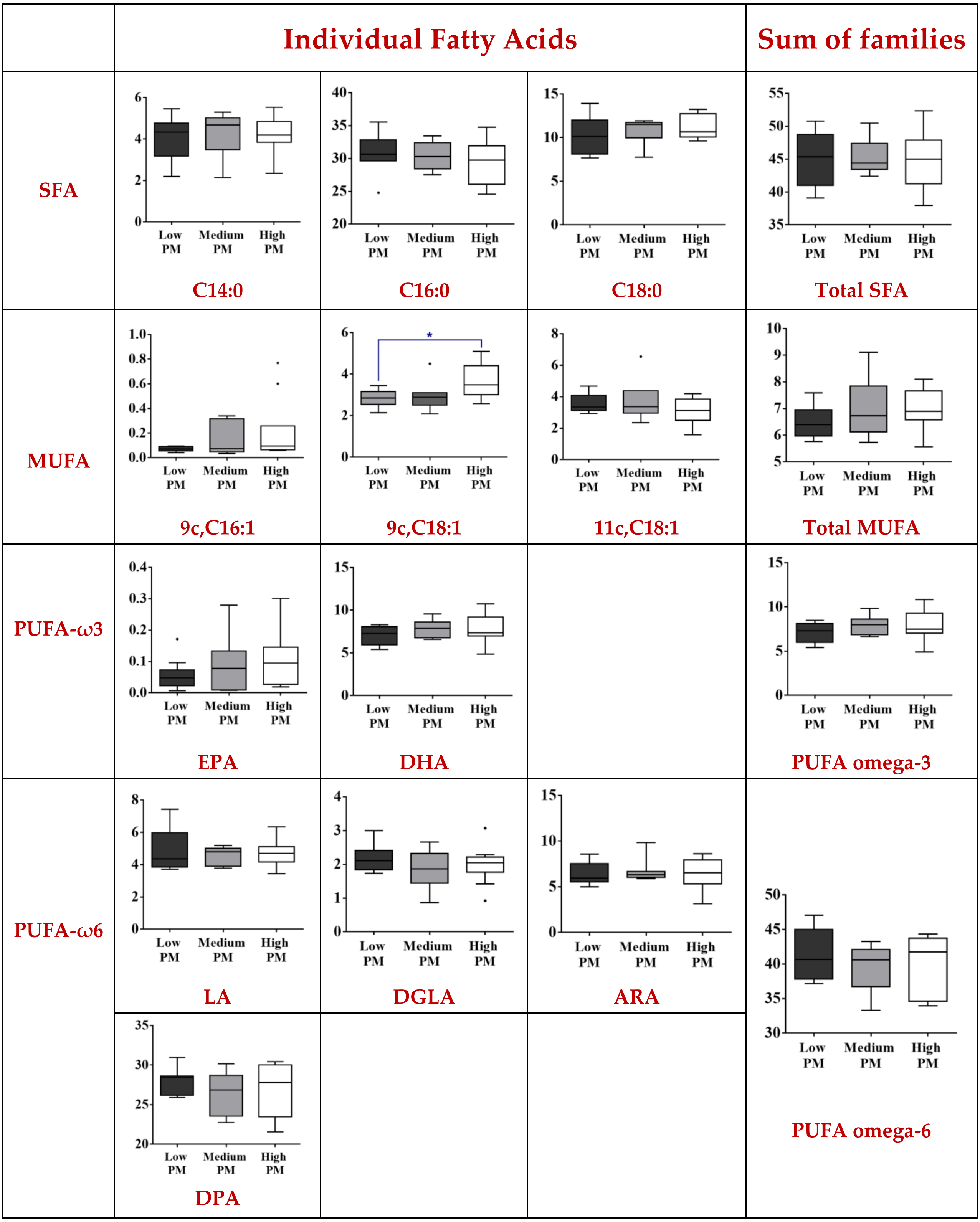

Supplementary Figure S8. Relative quantitative percentage differences between healthy donkeys with spermatozoa that demonstrated different levels of motility (%) for each fatty acid type and family: Low progressive Motility % (Dark Gray, Low PM<35%, n=9); Medium Progressive Motility % (Light Gray, 35%≤Medium PM<45%, n=7) and High Progressive Motility % (White, PM<45%, n=10) for each type of fatty acid in the spermatozoa membranes. The values are given as mean ± SD. Each member of the fatty acid family is given in a row, the last column being the sum of the corresponding fatty acid family. Values significantly different when compared to with each other: (*) $p < 0.05$. For donkey's' characteristics see Supplementary Table S1. For specific FA values see Table 2 in the main text.

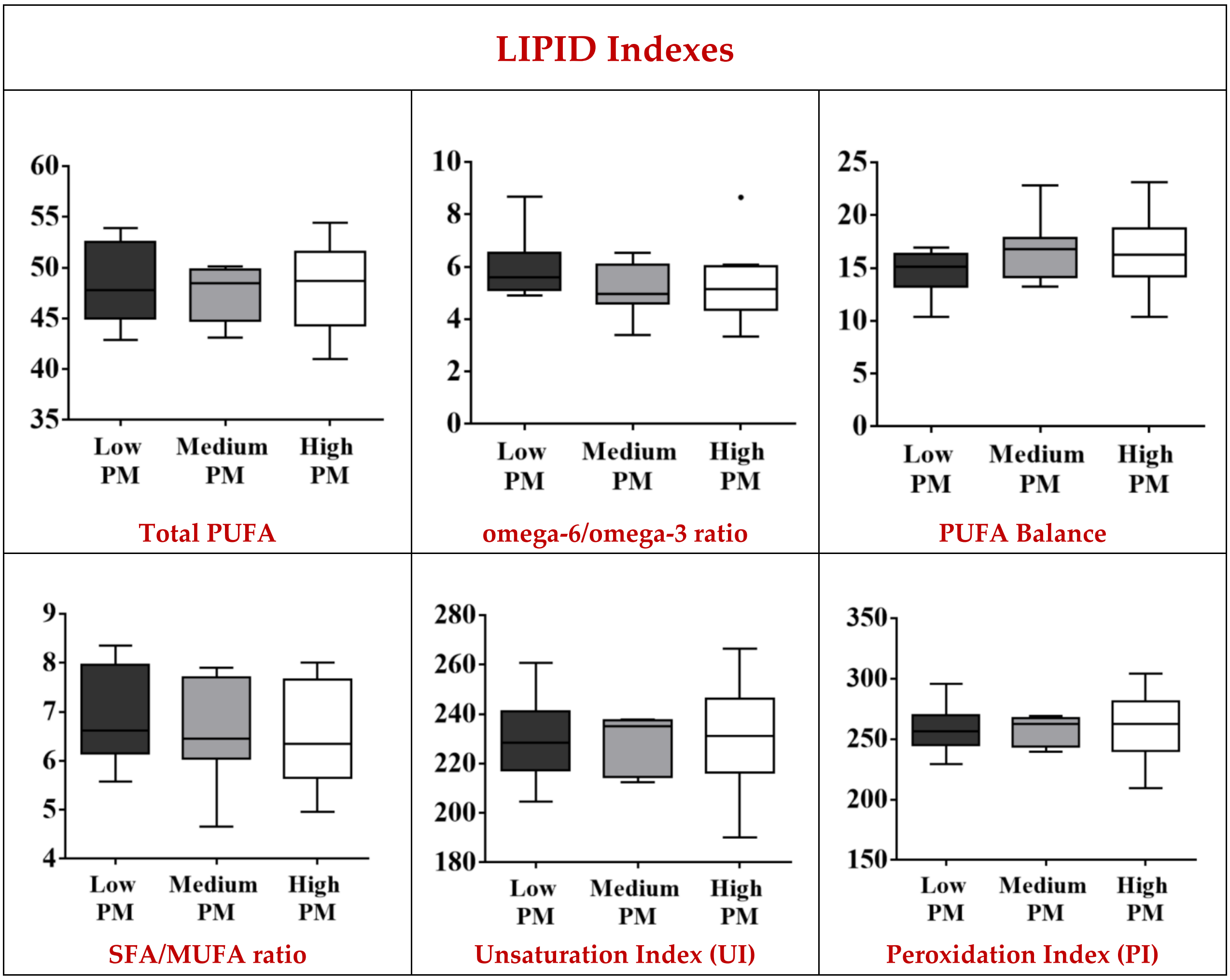

Supplementary Figure S9. Relative quantitative percentage differences between healthy donkeys with spermatozoa that demonstrated different levels of motility (%) for lipid indexes: Low progressive Motility % (Dark Gray, Low PM<35%, n=9); Medium Progressive Motility % (Light Gray, 35%≤Medium PM<45%, n=7) and High Progressive Motility % (White, PM<45%, n=10) for the membrane homeostasis indexes. 1st row: total PUFA, omega-6/omega-3 and SFA/PUFA ratios; 2nd row: SFA/MUFA ratio, unsaturation and peroxidation indexes. The values are given as mean ± SD. For donkey's' characteristics see Supplementary Table S1. For specific FA values see Table 2 in the main text.
